# Integrative Analysis Provides Insights into Genes Encoding LEA_5 Domain-Containing Proteins in Tigernut (*Cyperus esculentus* L.)

**DOI:** 10.3390/plants14050762

**Published:** 2025-03-01

**Authors:** Zhi Zou, Xiaowen Fu, Xiaoping Yi, Chunqiang Li, Jiaquan Huang, Yongguo Zhao

**Affiliations:** 1 National Key Laboratory for Tropical Crop Breeding/Hainan Key Laboratory for Biosafety Monitoring and Molecular Breeding in Off-Season Reproduction Regions, Institute of Tropical Biosciences and Biotechnology/Sanya Research Institute of Chinese Academy of Tropical Agricultural Sciences, Haikou 571101, China; xiaowen9924@126.com (X.F.); yixiaoping@itbb.org.cn (X.Y.); lichunqiang@itbb.org.cn (C.L.); 2 School of Breeding and Multiplication (Sanya Institute of Breeding and Multiplication) and College of Tropical Agriculture and Forestry, Hainan University, Sanya 572025, China; 3 College of Biology and Food Engineering, Guangdong University of Petrochemical Technology, Maoming 525000, China

**Keywords:** Cyperaceae, vegetative tissue, underground tuber, desiccation tolerance, late embryogenesis-abundant protein, phylogenomics

## Abstract

LEA_5 domain-containing proteins constitute a small family of late embryogenesis-abundant proteins that are essential for seed desiccation tolerance and dormancy. However, their roles in non-seed storage organs such as underground tubers are largely unknown. This study presents the first genome-scale analysis of the *LEA_5* family in tigernut (*Cyperus esculentus* L.), a Cyperaceae plant producing desiccation-tolerant tubers. Four *LEA_5* genes identified from the tigernut genome are twice of two present in model plants *Arabidopsis thaliana* and *Oryza sativa*. A comparison of 86 members from 34 representative plant species revealed the monogenic origin and lineage-specific family evolution in Poales, which includes the Cyperaceae family. *CeLEA5* genes belong to four out of five orthogroups identified in this study, i.e., LEA5a, LEA5b, LEA5c, and LEA5d. Whereas LEA5e is specific to eudicots, LEA5b and LEA5d appear to be Poales-specific and LEA5c is confined to families Cyperaceae and Juncaceae. Though no syntenic relationship was observed between *CeLEA5* genes, comparative genomics analyses indicated that LEA5b and LEA5c are more likely to arise from LEA5a via whole-genome duplication. Additionally, local duplication, especially tandem duplication, also played a role in the family expansion in *Juncus effuses*, *Joinvillea ascendens*, and most Poaceae plants examined in this study. Structural variation (e.g., fragment insertion) and expression divergence of *LEA_5* genes were also observed. Whereas *LEA_5* genes in *A. thaliana*, *O. sativa*, and *Zea mays* were shown to be preferentially expressed in seeds/embryos, *CeLEA5* genes have evolved to be predominantly expressed in tubers, exhibiting seed desiccation-like accumulation during tuber maturation. Moreover, *CeLEA5* orthologs in *C. rotundus* showed weak expression in various stages of tuber development, which may explain the difference in tuber desiccation tolerance between these two close species. These findings highlight the lineage-specific evolution of the *LEA_5* family, which facilitates further functional analysis and genetic improvement in tigernut and other species.

## 1. Introduction

Early methionine-labelled (EM)/D-19 proteins constitute a small family (LEA_5) of late embryogenesis abundant (LEA) proteins, which is defined by the presence of the conserved LEA_5 domain (PF00477) in the Pfam database [1]. This family was first characterized in wheat (*Triticum aestivum*) embryos [2], and then widely found in all three domains of life, i.e., eukaryotes, bacteria, and archaea [3,4,5]. LEA_5 family proteins are typical for the presence of approximately 20% Gly residues and a preponderance of charged and hydroxylated amino acids (AA), existing largely as random coils in solution and having a potential role in preventing freezing, desiccation, or osmotic stress damage [6,7,8]. In angiosperms, the family is usually present in two members, e.g., rice (*Oryza sativa*), maize (*Zea mays*), arabidopsis (*Arabidopsis thaliana*), papaya (*Carica papaya*), castor bean (*Ricinus communis*), and cassava (*Manihot esculenta*), which preferentially accumulate in embryonic tissues [9,10,11,12,13,14,15]. Their protein accumulation in seeds usually begins late in embryogenesis and the mRNA/protein abundances could be manipulated by abscisic acid (ABA) and osmotic stress [16,17,18]. In arabidopsis, both members (i.e., *AtLEA20*/*EM6* and *AtLEA35*/*EM1*) are required for normal seed development, and the *atem6-1* mutant was shown to be deficient in maturation drying [19,20]. Whereas *AtLEA35* predominantly accumulates in the vascular tissues and the meristem of the embryo, *AtLEA20* is expressed throughout the embryo as well as in non-seed organs [10,21,22]. Among two members present in rice, the transcripts of *OsLEA21* (*EM*/*EMP1*) at the seedling stage could be upregulated by ABA, cold, drought, and osmotic stresses, but downregulated by GA, salt, and flooded standing stresses [12]. Moreover, ABA modulation and seed-specific expression of *OsLEA21* was proven to be regulated by OsVP1, an ortholog of Viviparous-1 (VPl) in maize or ABA INSENSITIVE 3 (ABI3) in arabidopsis within the B3 transcription factor family, who works through binding the RY/Sph box or ABA-responsive element (ABRE) present in the promoter region [23,24,25].

Tigernut (*Cyperus esculentus* L. var. *sativus* Baeck.) is an oil-bearing tuber plant that belongs to the Cyperaceae family within the monocot clade [26,27,28]. Though it originated in the Mediterranean coast, tigernut is emerging as a novel oil crop widely cultivated in tropical, subtropical, and temperate zones for its potential health benefits, high biomass, and wide adaptability [29,30,31,32]. Unlike other tubers (e.g., potato (*Solanum tuberosum*)) and tuberous roots (e.g., cassava) that are highly sensitive to desiccation, the water content of mature tigernut tubers can drop to less than 6% without affecting the sprouting capability, which is comparative to orthodox seeds [33,34,35]. Correspondingly, comparative proteomic analysis revealed a seed-like proteome of mature tubers, including significant accumulations of oleosins, caleosins, as well as LEA proteins [34,35,36,37,38]. Among various LEA proteins, only seed maturation protein (SMP) and LEA_1 families have been systematically characterized in tigernut, which were shown to exhibit seed desiccation-like accumulation during tuber maturation [35,38]. Given the essential roles of LEA_5 proteins in desiccation tolerance, in this study, we would like to report a genome-wide identification, evolutionary, and expression analyses of the *LEA_5* gene family in tigernut, which revealed the monogenic origin and lineage-specific family evolution in Poales, including the Cyperaceae family. Moreover, our results imply that the desiccation tolerance of tigernut tubers is more likely to be contributed by tuber-specific activation of *LEA_5* genes by certain transcription factors different from those of orthodox seeds. These findings will provide valuable information for further functional analyses.

## 2. Results

### 2.1. Characterization of Four LEA_5 Family Genes in Tigernut

**Figure 1:**
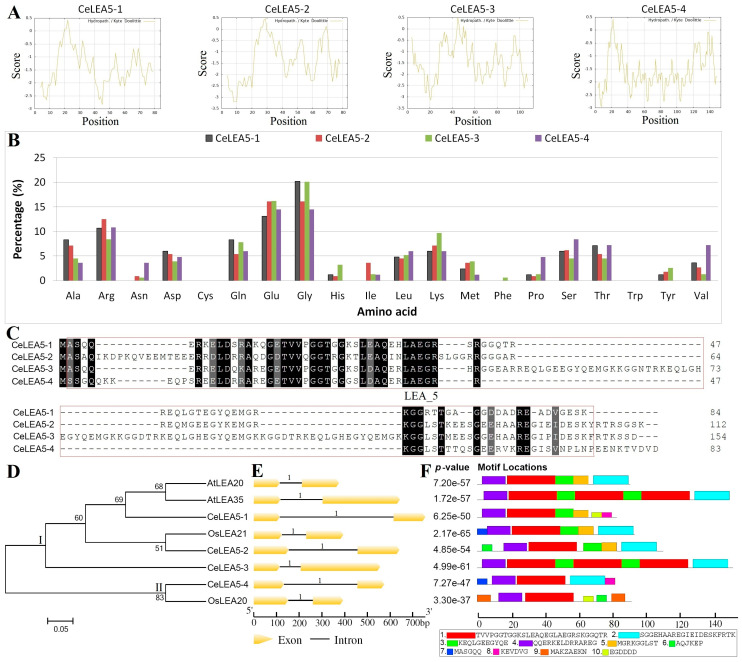
Structural and phylogenetic analyses of *LEA_5* family genes in *C. esculentus*. (**A**) Kyte–Doolittle hydrophobicity plots of CeLEA5 proteins using ProtScale (v1). (**B**) Amino acid composition of CeLEA5 proteins. (**C**) Multiple sequence alignment of CeLEA5 proteins using MUSCLE (v5.1). Identical and similar amino acids are highlighted in black or dark grey, respectively, whereas conserved LEA_5 domains are boxed in red. (**D**) An unrooted phylogenetic tree resulting from full-length Ce/Os/AtLEA5 proteins with RAxML (maximum likelihood method and bootstrap of 1000 replicates), where the distance scale denotes the number of amino acid substitutions per site. The name of each clade (i.e., I and II) is indicated next to the corresponding group. (**E**) The exon-intron structures. “1” represents the intron phase that is located between the first and second bases of a codon. (**F**) The distribution of conserved motifs among Ce/Os/AtLEA5 proteins, where different motifs are represented by different color blocks as indicated and the same color block in different proteins indicates a certain motif. (At: *A. thaliana*; Ce: *C. esculentus*; LEA: Late embryogenesis abundant; Os: *Oryza sativa*).

As shown in Table 1, a total of four genes that encode LEA_5 domain-containing proteins were identified from four scaffolds (Scfs) of the tigernut genome, and all of them were detected in the full-length transcriptome as described before [39], supporting their expression and putative functions. Their CDS (coding sequence) length varies from 252 to 465 bp (base pair), which was predicted to encode 83–154 AA with the MW (molecular weight) of 8.88–16.92 kDa (kilodalton) (Table 1). Though the overall sequence similarity of deduced proteins varies from 36.20% to 59.82% (Table S1), all of them harbor at least one LEA_5 domain, i.e., one for CeLEA5-1 and two for others (Table 1). The theoretical pI (isoelectric point) values were shown to range from 5.22 to 6.02, and the GRAVY (grand average of hydropathicity) values are between −1.313 to −1.582 (Table 1), implying the acidic and hydrophilic features. The hydrophilic feature was also supported by the ProtScale analysis, which revealed similar hydropathicity scales for all four CeLEA5 proteins (Figure 1A). Comparing the AA composition showed that, except for Cys and Trp, other 18 AA were found in at least one of four proteins, which are rich in Gly, Glu, and Arg. Additionally, Phe is only present in CeLEA5-3, whereas Asn and His/Tyr are absent from CeLEA5-1 and -4, respectively (Figure 1B). Compared with CeLEA5-1 and -4, sequence alignment revealed that the longer protein length of CeLEA5-2 and -3 was caused by fragment insertion (Figure 1C).

It is worth noting that the family amounts in tigernut are twice of two present in two well-studied model plants arabidopsis and rice (Table S2), implying species or lineage-specific expansion. To disclose their evolutionary relationships, an unrooted phylogenetic tree was constructed using full-length protein sequences of eight *LEA_5* genes in these three species. As shown in Figure 1D, these proteins were clustered into two main groups. Notably, AtLEA20 and -35, which share 53.59% sequence similarity, were clustered in species in Group I. By contrast, OsLEA21 was clustered with CeLEA5-2, exhibiting 72.32% sequence similarity, which is slightly smaller than 77.89% observed between OsLEA21 and CeLEA5-1. Group II includes CeLEA5-4 and OsLEA20, which exhibit 61.70% sequence similarity (Table S1).

To uncover possible structural variation, gene structures and conserved motifs were further compared. In contrast to the invariable exon-intron structure with a single intron in phase 1 (Figure 1E), our MEME analysis revealed distinct motif composition among Ce/Os/AtLEA5 proteins. Among the 10 motifs identified as shown in Figure 1F, Motifs 1 and 4 are highly conserved and shared by all sequences. Whereas Motif 4 is present in a single copy, Motif 1 appears in three copies in both CeLEA5-3 and AtLEA35, implying tandem duplication. Motifs 2, 3, and 5 are also widely distributed, which are only absent from CeLEA5-1/OsLEA20, CeLEA5-4/OsLEA20, and CeLEA5-3/CeLEA5-4/OsLEA20, respectively. Like Motif 1, Motif 3 appears in two copies in both CeLEA5-3 and AtLEA35. By contrast, other motifs are sequence-specific. Whereas Motif 9 is confined to OsLEA20 (in two copies), Motifs 6, 7, 8, and 10 are only present in CeLEA5-2/OsLEA20, CeLEA5-4/OsLEA21, CeLEA5-1/-4, and CeLEA5-1/OsLEA20, respectively (Figure 1F). Notably, except for Motifs 6, 7, 8, and 9, other motifs belong to the LEA_5 domain (Figure 1F), implying possible functional divergence.

### 2.2. Characterization of LEA_5 Genes from Representative Plant Species and Insights into Lineage-Specific Family Evolution

Since the origin and evolution of *CeLEA5* genes were not well resolved by above phylogenetic analysis, a similar approach was also used to identify homologs from 33 representative plant species, which include the basal angiosperm *Amborella trichopoda*, four core eudicots, 25 core monocots, and three early diverged monocots that did not experience the τ WGD, i.e., *Acorus gramineus*, eelgrass (*Zostera marina*), and duckweed (*Spirodela polyrhiza*) [40,41]. As shown in Table S2, a single member was not only identified in *A. trichopoda*, a rare example without any recent whole-genome duplication (WGD), but also in eelgrass, duckweed, apostasia (*Dendrobium catenatum*), and *Dioscorea alata*, though they were proven to have undergone at least one recent WGD after monocot radiation [40,41,42]. By contrast, 2–6 members were found in other species (Table S2), implying the monogenic origin of the *LEA_5* family followed by lineage-specific expansion.

**Figure 2:**
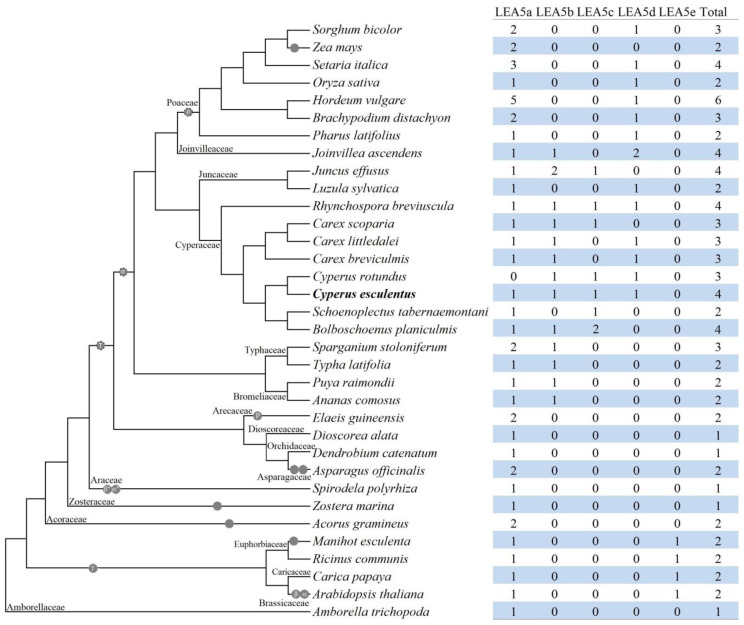
Species-specific distribution of five orthogroups in 34 representative plant species. The species tree is referred to NCBI Taxonomy (https://www.ncbi.nlm.nih.gov/taxonomy, accessed on 20 November 2024) and well-established recent WGDs are marked: γ represents the whole-genome triplication event shared by all core eudicots; β and α represent two WGDs that are specific to Brassicaceae; β″ and α″ represent two Araceae-specific WGDs; τ represents the WGD shared by all core monocots; p represents the Arecaceae-specific WGD; σ represents the Poales-specific WGD; and ρ represents the Poaceae-specific WGD. Names of tested plant families are indicated next to the corresponding branches. (LEA: Late embryogenesis abundant; WGD: whole-genome duplication).

To infer lineage-specific evolution, orthologous genes among different species were clustered using Orthofinder [43]. As shown in Figure 2 and Table S3, a total of five orthogroups were identified. Whereas LEA5a is shared by both monocots and eudicots, LEA5b/LEA5c/LEA5d and LEA5e are specific to monocots and eudicots, respectively. *LEA_5* family genes in *A. trichopoda*, *A. gramineus*, eelgrass, duckweed, garden asparagus (*Asparagus officinalis*), and oil palm (*Elaeis guineensis*) belong to LEA5a, which also includes CeLEA5-1 and OsLEA21 (Table S3), though OsLEA21 was grouped with CeLEA5-2 in the phylogenetic tree as shown in Figure 1D. LEA5b and LEA5d appear to be Poales-specific, whereas LEA5c seems to be confined to Cyperaceae and Juncaceae (Table S3).

**Figure 3:**
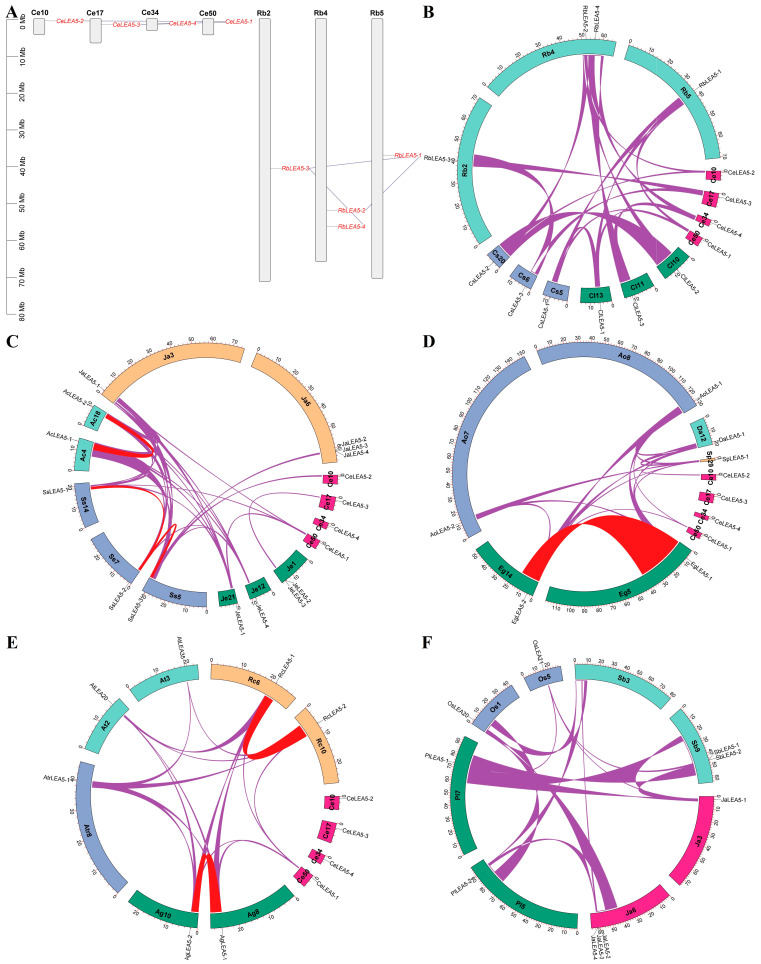
Synteny analyses within and between *C. esculentus* and representative plant species. (**A**) Chromosomal localization and duplication events of the *LEA_5* family genes in *C. esculentus* and *R. breviuscula*. (**B**) Synteny analyses within and between *C. esculentus*, *C. littledalei*, *C. scoparia*, and *R. breviuscula*. (**C**) Synteny analyses within and between *C. esculentus*, *J. effusus*, *S. stoloniferum*, *A. comosus*, and *J. ascendens*. (**D**) Synteny analyses within and between *C. esculentus*, *E. guineensis*, *A. officinalis*, and *D. alata*. (**E**) Synteny analyses within and between *C. esculentus*, *A. gramineus*, *A. trichopoda*, *A. thaliana*, and *R. communis*. (**F**) Synteny analyses within and between *J. ascendens*, *P. latifolius*, *O. sativa*, and *S. bicolor*. Shown are *LEA_5* gene-encoding chromosomes/scaffolds and only syntenic blocks containing *LEA_5* genes are marked, where red and purple lines indicate intra- and inter-species, respectively. The scale is in Mb. (Ac: *A. comosus*; Ag: *A. gramineus*; Ao: *A. officinalis*; At: *A. thaliana*; Atr: *A. trichopoda*; Bd: *B. distachyon*; Ce: *C. esculentus*; Cl: *C. littledalei*; Cs: *C. scoparia*; *Da*: *D. alata*; Eg: *E. guineensis*; Ja: *J. ascendens*; Je: *J. effuses*; Mb: megabase; Os: *O. sativa*; *Pl: P. latifolius*; Rb: *R. breviuscula*; Rc: *R. communis*; Sb: *S. bicolor*; Ss: *S. stoloniferum*).

To gain insights into the origin of *LEA_5* genes, species-specific duplication events were further examined. As shown in Figure 3A, no syntenic relationship was observed between *CeLEA5* genes. Instead, *CeLEA5-2*/*-3* and *-4* were characterized as dispersed repeats of *CeLEA5-1* and *-3*, respectively, which is similar to that observed in *Rhynchospora breviuscula*, another Cyperaceae plant. Further interspecific synteny analyses revealed that all *CeLEA5* genes have syntelogs in at least one out of 32 species tested in this study, which includes papaya, castor bean, cassava, *A. gramineus*, and duckweed. Significantly, 1:1 syntenic relationships were observed between tigernut and *R. breviuscula*, providing direct evidence of early divergence into four groups before Cyperaceae radiation (Figure 3B). Moreover, *CeLEA5-1* harbors syntelogs not only in *Juncus effuses* (a Juncaceae plant within Poales), *Sparganium stoloniferum* (a Typhaceae plant within Poales), and *Joinvillea ascendens* (a Joinvilleaceae plant within Poales) (Figure 3C), but also in oil palm, garden asparagus (Figure 3D), *A. gramineus*, and castor bean (Figure 3E), whereas *CeLEA5-2* and *-3* have syntelogs in *S. stoloniferum*/duckweed and *J. effuses*, respectively (Figure 3C,D). The location of *SsLEA5-1*, *-2*, and *-3* within syntenic blocks provides direct evidence of LEA5b from LEA5a via WGD, most likely the σ event shared by all Poales plants [44], followed by species-specific expansion via WGD in *S. stoloniferum* [45]. Notably, though no syntelog was identified for *DaLEA5-1* in tigernut, *D. alata* exhibits 1:1, 1:2, with 1:3 syntenic relationships with duckweed, *J. effuses*/*J. ascendens*, and *S. stoloniferum*, respectively, providing direct evidence of LEA5c from LEA5a via WGD (Figure S1A). Interestingly, in contrast to no syntelog that was identified for all four *CeLEA5* genes in *A. trichopoda* and arabidopsis, *A. trichopoda* exhibits 1:1 and 1:2 syntenic relationships with arabidopsis/castor bean and *A. gramineus* (Figure 3E). Moreover, though *AtLEA20* and *-35* were characterized as dispersed repeats, both of them were shown to be located within syntenic blocks with *CpLEA5-1*, *CpLEA5-2*, *RcLEA5-1*, *RcLEA5-2*, *MeLEA5-1*, and *MeLEA5-2* (Figure S1B), implying their WGD-derivation followed by species-specific chromosome rearrangement. Interestingly, in contrast to no syntelog that was identified for *CeLEA5* genes in all tested Poaceae species, both *JaLEA5-1* and *-3* were shown to have syntelogs in these species, e.g., *Pharus latifolius*, rice, and sorghum (*Sorghum bicolor*). Additionally, tandem duplication was also shown to play a key role in gene expansion of the *LEA_5* family in Poales, e.g., *J. effuses*, *J. ascendens*, *Brachypodium distachyon*, barley (*Hordeum vulgare*), foxtail millet (*Setaria italica*), and sorghum (Table S2). Notably, despite the occurrence of one additional WGD after the split with sorghum [46], maize has two LEA5a members that were characterized as proximal repeats (Table S2).

### 2.3. CeLEA5 Genes Have Evolved to Be Preferentially Expressed in Tigernut Tubers

To provide a global view of the expression evolution of *LEA_5* genes, the tissue-specific expression profiles were first mined from the Plant Public RNA-seq Database, which includes 28,164, 11,726, and 19,664 libraries for arabidopsis, rice, and maize, respectively. As shown in Figure S2, all of them exhibit a seed/embryo-preferential expression pattern.

**Figure 4:**
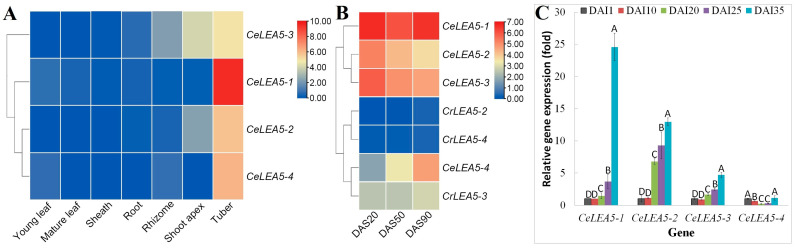
Expression profiles of *Ce/CrLEA5* genes. (**A**) Tissue-specific expression profiles of five *CeLEA5* genes. (**B**) Expression profiles of *Ce/CrLEA5* genes at three representative stages of tuber development. (**C**) Expression profiles of *CeLEA5-1*, *-2*, *-3*, and *-4* at different stages of tuber development. The heatmap was generated using the R package (v2) implemented with a row-based standardization. Color scale represents FPKM normalized log_2_ transformed counts, where blue indicates low expression and red indicates high expression. Bars indicate SD (N = 3) and uppercase letters indicate difference significance tested following Duncan’s one-way multiple-range post hoc ANOVA (*p* < 0.01). (Ce: *C. esculentus*; Cr: *C. rotundus*; DAI: days after tuber initiation; DAS: days after sowing; FPKM: Fragments per kilobase of exon per million fragments mapped).

Global expression profiles of *CeLEA5* genes were examined in seven main tissues/developmental stages, i.e., shoot apex, young leaf, mature leaf, leaf sheath, root, rhizome, and tuber. Interestingly, all four *CeLEA5* genes were shown to be predominantly expressed in tubers (Figure 4A), which represent the maturation stage of 120 days after sowing (DAS). Among them, *CeLEA5-1* transcripts were most abundant, followed by *CeLEA5-4* and *-2*, and least for *CeLEA5-3*. Both *CeLEA5-1* and *-4* were barely expressed in other tissues, whereas *CeLEA5-2* and *-3* were also expressed in shoot apexes and/or rhizomes, though their transcript levels were considerably lower than those in tubers (Figure 4A). The results support expression and possible functional divergence of *CeLEA5* genes and between paralogs.

### 2.4. CeLEA5 Genes Were Expressed More than Their Orthologs in C. rotundus

The absence of a *CeLEA5-1* (the dominant member) ortholog in the tuber transcriptome assembly of purple nutsedge (*C. rotundus*) (Table S2), a desiccation-sensitive tuber plant close to tigernut, implies a possible less important role of *LEA_5* genes in the tuber development of this species. To test this hypothesis, expression profiles of *LEA_5* genes in three representative stages of tuber development were compared, i.e., 20, 50, and 90 DAS, which represent tuber initiation, swelling, and maturation, respectively. As shown in Figure 4B, except for *CrLEA5-3*, the transcripts of the other two *CrLEA5* genes were rarely detected. Moreover, *CrLEA5-3* transcripts were considerably lower than those of all four *CeLEA5* genes (Figure 4B), implying species-specific activation of *LEA_5* genes in tigernut tubers, especially at the maturation stage.

### 2.5. Transcripts of Most CeLEA5 Genes Were Gradually Upregulated During Tuber Development

To learn more about the expression profiles of *CeLEA5* genes during tuber development, five typical stages were examined using qRT-PCR, i.e., 1, 10, 20, 25, and 35 days after tuber initiation (DAI), which represent initiation, early swelling, middle swelling, late swelling, and maturation, respectively [28]. The moisture content of the first three stages was characterized as approximately 85%, and the two latter stages were about 75% and 48%, respectively [35]. As shown in Figure 4C, a gradual increase in transcripts during tuber development was observed for *CeLEA5-1*, *-2*, and *-3*, though no significant difference was observed between the two early stages, i.e., 10 vs. 1 DAI. By contrast, *CeLEA5-4* transcripts were gradually downregulated at three swelling stages, but also peaked at the maturation stage as other members (Figure 4C), implying their putative roles in the acquisition of desiccation tolerance.

## 3. Discussion

Desiccation tolerance, an ancient adaptation trait appearing very early in the evolution of terrestrial life, is usually present in spores, pollen, and seeds of vascular plants [47]. The underlying mechanism is usually associated with significant accumulation of LEA proteins, a class of small and extremely hydrophilic proteins that were first discovered in cotton (*Gossypium hirsutum*) seeds [22]. Among them, LEA_5 domain-containing proteins constitute a small family (usually in two copies) that are essential for stress responses as well as desiccation tolerance and dormancy in orthodox seeds [19,20,22,48]. However, their roles in non-seed storage organs are largely unknown.

### 3.1. The Tigernut Genome Encodes a High Number of Four LEA_5 Genes, and the Family Expansion Was Contributed by Dispersed Duplication

Tigernut is a unique plant producing desiccation-tolerant tubers, which differs from its relative purple nutsedge, another Cyperaceae plant bearing desiccation-sensitive tubers [34,38]. In this study, the first genome-wide characterization of the *LEA_5* gene family was conducted in tigernut, and a high number of four members with one to two conserved LEA_5 domains were obtained. Interestingly, all of them were detected in transcriptomes from both Illumina RNA-seq and PacBio Single-Molecule Real-Time (SMRT) sequencing [39]. By contrast, only three members corresponding to *CeLEA5-2*, *-3*, and *-4* were identified from the transcriptome *de novo* assembled from Illumina RNA-seq of purple nutsedge tubers [36]. Notably, similar to arabidopsis and rice, four *CeLEA5* genes were also shown to be dispersed repeats.

### 3.2. Comparative Genomics Analysis Reveals the Monogenic Origin and Lineage-Specific Evolution of the LEA_5 Family in Poales

Tigernut resides in the Cyperaceae family within the Poales order, which also includes the well-known Poaceae family [27,49]. It was proven that after the monocot-eudicot split, all core eudicots shared one so-called γ WGT (whole-genome triplication), whereas all monocots with the exception of Acorales and Alismatales plants (also called core monocots) experienced the τ WGD [44,50]. Furthermore, Poales plants underwent one order-specific σ WGD, and all Poaceae plants shared the ρ WGD [44].

To learn more about the origin and evolution of *CeLEA5* genes, a total of 82 homologs were further identified from 33 representative plant species, which belong to 17 plant families, i.e., Amborellaceae (1), Brassicaceae (1), Caricaceae (1), Euphorbiaceae (2), Acoraceae (1), Zosteraceae (1), Araceae (1), Asparagaceae (1), Orchidaceae (1), Dioscoreaceae (1), Arecaceae (1), Bromeliaceae (2), Typhaceae (2), Cyperaceae (7), Juncaceae (2), Joinvilleaceae (1), and Poaceae (7). The data cover the majority of genome-available plant families within the lineage of core monocots, where Acoraceae/Zosteraceae/Araceae (early diverged monocots), Brassicaceae/Caricaceae/Euphorbiaceae (core eudicots), and Amborellaceae (the basal angiosperm) were used as out-groups for core monocots, monocots, and core angiosperms, respectively. A single member found in *A. trichopoda*, eelgrass (an early diverged monocot in Alismatales), and duckweed (another early diverged monocot in Alismatales) implies the monogenic origin of this gene family in angiosperms. Correspondingly, *LEA_5* genes in these species could be assigned into the same orthogroup, i.e., LEA5a. Moreover, though *A. gramineus* (an early diverged monocot in Acorales) has two members, both of which belong to LEA5a and were characterized as recent WGD repeats, which is similar to oil palm whose family expansion was contributed by the Arecaceae-specific p WGD [51]. By contrast, four *CeLEA5* genes were assigned into four out of five orthogroups identified in this study, i.e., LEA5a, LEA5b, LEA5c, and LEA5d. Unlike LEA5a, which is shared by both monocots and eudicots, LEA5e is specific to eudicots, whereas the other three groups appear to be monocot-specific. More exactly, both LEA5b and LEA5d are more likely to be Poales-specific, while LEA5c is limited to Cyperaceae and Juncaceae. Though the origin of LEA5d was not well resolved, both LEA5b and LEA5c are more likely to arise from WGD, because *DaLEA5-1*, *SsLEA5-1*, *SsLEA5-2*, *SsLEA5-3*, *JaLEA5-1*, *JaLEA5-2*, *JeLEA5-1*, and *JeLEA5-4* are still located within syntenic blocks. Whereas LEA5b is more likely to be generated by the σ WGD, LEA5c may be derived from the WGD as described in *C. littledalei* [52]. That means that both *CeLEA5-2* and *-3* are more likely to arise from *CeLEA5-1* via WGD followed by chromosome rearrangement. Since no syntenic relationship was observed between *LEA_5* genes present in all species examined in both Cyperaceae and Juncaceae, the rearrangement is more likely to occur sometime before the split between these two families. On the contrary, the rearrangement between *AtLEA20* and *-35* may be species-specific, since their orthologs in papaya, castor bean, and cassava are still located within syntenic blocks.

Besides WGD, local duplication such as tandem and proximal duplications also played a role in the family expansion, especially in Poaceae plants, varying from two to five copies. Additionally, gene contraction was also frequently observed and a good example is the Juncaceae family. Among two species examined in this family, *J. effusus* has four members that belong to LEA5a (1), LEA5b (2), and LEA5c (1), whereas *Luzula sylvatica* harbors only two members that belong to LEA5a (1) and LEA5d (1). However, the biological significance needs to be further studied.

### 3.3. CeLEA5 Genes Underwent Apparent Expression and Functional Divergence

Seed-preferential expression of *LEA_5* genes has been documented in a high number of plant species, including wheat, maize, rice, barley, arabidopsis, castor bean, and papaya [9,10,11,13,14,22]. Correspondingly, our large-scale transcriptional profiling conducted in arabidopsis, rice, and maize indeed supports a seed/embryo-predominant expression pattern of *LEA_5* genes. By contrast, four *CeLEA5* genes were shown to have evolved to be predominantly expressed in the tigernut tubers, implying their neofunctionalization in vegetative tissues. Interestingly, their orthologs in purple nutsedge showed low expression in the tuber transcriptomes of three representative developmental stages, i.e., 20, 50, and 90 DAS. Since purple nutsedge tubers are highly sensitive to desiccation [34], species rather than tuber-specific activation of *LEA_5* genes in tigernut could be speculated. The distinct patterns may explain the difference in tuber desiccation tolerance between these two close species. Moreover, during tuber development, except for *CeLEA5-4*, gradual upregulation of other *CeLEA5* genes since 10 DAI was observed, which is negatively correlating with the dynamics of water content [27,35]. Sharp transcript increase for all four *CeLEA5* genes during tuber maturation is highly similar to that observed in orthodox seeds, which is accompanied by the acquisition of desiccation tolerance and dormancy [20,47]. It has been suggested that LEA_5 proteins could replace water and thus protect the embryo during the phase of desiccation [53]. A similar role of CeLEA5 proteins in tigernut tubers could be speculated. Further gene knockout and overexpression of *CeLEA5* genes in purple nutsedge and potato tubers may provide more evidence. Additionally, despite the central role of ABI3/VP1 in regulating *LEA_5* genes in seeds [23,24,25,54], a distinct regulatory mechanism may be present for *CeLEA5* genes, since no evidence is available for the expression of an *ABI3/VP1* homolog (CESC_01781) in tigernut tubers as well as other tissues with available transcriptome data. Moreover, we could not isolate its mRNA by using the PCR technology. Thereby, further identifying transcription factors that mediate tuber-specific activation of *CeLEA5* genes is of particular interest.

## 4. Conclusions

This study presents the first genome-scale analysis of the *LEA_5* gene family in tigernut, a Cyperaceae plant producing desiccation-tolerant tubers. Identification and comparison of 86 members from 34 plant species representing 17 plant families support the monogenic origin in angiosperms and lineage-specific family evolution in Poales. Though four *CeLEA5* genes were characterized as dispersed repeats, they belong to four out of five orthogroups, which were derived from WGD (LEA5b, LEA5c, and LEA5e) and dispersed duplication (LEA5d). Whereas LEA5e is specific to eudicots, LEA5b and LEA5d appear to be Poales-specific, and LEA5c is limited to Cyperaceae and Juncaceae. In contrast to seed/embryo-preferential expression of *LEA_5* genes in other species, *CeLEA5* genes have evolved to be predominantly expressed in tubers, exhibiting seed desiccation-like accumulation during tuber maturation. These findings highlight the lineage-specific evolution of the *LEA_5* family and putative roles of *CeLEA5* genes in acquiring desiccation tolerance of tigernut tubers, which facilitates further functional analysis and genetic improvement in tigernut and other species.

## 5. Materials and Methods

### 5.1. Identification of LEA_5 Family Genes from Datasets

Genome sequences of representative plant species were downloaded from Genome Warehouse (https://ngdc.cncb.ac.cn/gwh/, accessed on 20 November 2024), TAIR11 (https://www.arabidopsis.org/, accessed on 20 November 2024), Phytozome v13 (https://phytozome.jgi.doe.gov/pz/portal.html, accessed on 20 November 2024), and NCBI (https://www.ncbi.nlm.nih.gov/, accessed on 20 November 2024): *A. trichopoda* (v2.1), *A. thaliana* (Araport11), *C. papaya* (Sunset), *R. communis* (WT05), *M. esculenta* (v8), *A. gramineus* (v1), *Z. marina* (v3.1), *S. polyrhiza* (v2), *D. alata* (v1), *D. catenatum* (v1), *A. officinalis* (v1.1), *E. guineensis* (v3), *A. comosus* (v3), *Puya raimondii* (v1), *S. stoloniferum* (v1), *Typha latifolia* (v1), *O. sativa* (v7.0), *H. vulgare* (Morex V3), *B. distachyon* (v3.2), *S. italica* (v2.2), *S. bicolor* (v5.1s), *Z. mays* (RefGen_V4), *C. esculentus* (v1), *C. littledalei* (v1), *C. breviculmis* (v1), *C. scoparia* (v1), *S. tabernaemontani* (v1), and *B. planiculmis* (v1). Transcriptome data of tigernut were accessed from NCBI (https://www.ncbi.nlm.nih.gov/, accessed on 20 November 2024), whereas for purple nutsedge, the *de novo* assembled transcriptome described before [36] was adopted. To identify *LEA_5* family genes, HMMER (v3.3.2, http://hmmer.janelia.org/, accessed on 20 November 2024) searches were performed using the Pfam profile PF03760 (v35.0, http://pfam.xfam.org/, accessed on 20 November 2024). Gene models of candidates were manually revised with mRNAs when available, whereas gene structures were displayed using GSDS 2.0 [55]. The presence of the conserved LEA_5 domain in deduced proteins was confirmed using Pfam Search (http://pfam.xfam.org/, accessed on 20 November 2024), and biochemical parameters were calculated using ProtParam (http://web.expasy.org/protparam/, accessed on 20 November 2024). Expression data of arabidopsis, rice, and maize were accessed from the Plant Public RNA-seq Database (http://plantrnadb.com/, accessed on 20 November 2024).

### 5.2. Phylogenetic and Conserved Motif Analyses

Multiple sequence alignments were carried out using Muscle v5.1 [56], and phylogenetic tree construction was performed using RAxML (http://www.phylo.org/portal2/home.action#, accessed on 20 November 2024) with the maximum likelihood method and bootstrap of 1000 replicates. Conserved motifs were identified using MEME (v5.4.1, http://meme-suite.org/tools/meme, accessed on 20 November 2024) with the parameters as follows: any number of repetitions; the maximum number of motifs, 10; and, the optimum width of each motif, between 5 and 50 residues.

### 5.3. Synteny Analysis and Definition of Orthogroups

Orthologous genes were clustered using Orthofinder (v2.3.8) [43]. Synteny analysis was conducted as previously described [57], and gene duplication modes were identified using the DupGen_finder pipeline [58].

### 5.4. Plant Materials

The growing conditions of tigernut plants (Reyan3) were as previously described [27]. Tubers were collected at 1, 10, 20, 25, and 35 DAI, which represent tuber initiation, three stages of swelling (early, middle, and late), and maturation as described before [28]. Once collected, samples with three biological replicates were frozen with liquid nitrogen and stored at −80 °C for further use.

### 5.5. Gene Expression Analysis Based on RNA-Seq

Global expression profiles of *Ce/CrLEA5* genes were analyzed using the Illumina RNA-seq datasets PRJNA703731 and PRJNA671562, which are 150 bp paired-end reads with three biological replicates. Except for mature tubers that were collected at 120 DAS, other tissues (i.e., shoot apex, young leaf, mature leaf, sheath of mature leaf, root, and rhizome) were collected at 85 DAS. Different stages of tuber development in tigernut and purple nutsedge were collected at 20, 50, and 90 DAS, respectively. Quality control of raw RNA-seq reads and subsequent read mapping were conducted as previously described [48], and the relative gene expression level was presented as FPKM [59].

### 5.6. Gene Expression Analysis Based on qRT-PCR

Total RNA extraction, integrity and concentration detection, and synthesis of the first-strand cDNA were carried out as described before [27,35]. Primers used for qRT-PCR analysis are shown in Table S3, where *CeTIP41* and *CeUCE2* [27] are two reference genes. PCR reaction, relative gene abundance calculation, and statistical analysis were conducted as previously described [60], where differences among means were tested following Duncan’s one-way multiple-range post hoc ANOVA in Data Processing System software v20.

## Figures and Tables

**Table 1:** *LEA_5* family genes identified in *C. esculentus*. (AA: amino acid; Ce: *C. esculentus*; GRAVY: grand average of hydropathicity; kDa: kilodalton; LEA: Late embryogenesis abundant; MW: molecular weight; pI: isoelectric point; Scf: scaffold).

Gene Name	Locus ID	Position	AA	MW (kDa)	pI	GRAVY	LEA_5 Location	Duplicate	Mode
*CeLEA5-1*	CESC_21264	Scf50:743435..744190(−)	84	8.88	5.43	−1.398	2..67	-	
*CeLEA5-2*	CESC_23041	Scf10:593714..594313(−)	112	12.34	5.61	−1.388	16..58, 57..105	*CeLEA5-1*	Dispersed
*CeLEA5-3*	CESC_08006	Scf17:1581661..1582217(+)	154	16.92	6.02	−1.582	2..84, 82..148	*CeLEA5-1*	Dispersed
*CeLEA5-4*	CESC_16081	Scf34:1210271..1210837(−)	83	8.96	5.22	−1.313	9..49, 47..70	*CeLEA5-3*	Dispersed

## Data Availability

Transcriptome data used in this study is under the NCBI accession numbers PRJNA703731 and PRJNA671562.

## Supplementary Materials

The following supporting information can be downloaded at: https://www.mdpi.com/article/10.3390/plants14050762/s1, Figure S1: Synteny analyses within and between between *D. alata/J. effuses/S. stoloniferum/J. ascendens* (A) and *A. thaliana/C. papaya/R. communis/M. esculenta* (B). Shown are *LEA_5* gene-encoding chromosomes/scaffolds and only syntenic blocks containing *LEA_5* genes are marked, where red and purple lines for intra- and inter-species, respectively. The scale is in Mb. (At: *A. thaliana*; Cp: *C. papaya*; Da: *D. alata*; Ja: *J. ascendens*; Je: *J. effuses*; LEA: late embryogenesis-abundant; Mb: megabase; Me: *M. esculenta*; Rc: *R. communis*; Ss: *S. stoloniferum*). Figure S2: Global expression profiles of *AtLEA5*, *OsLEA5*, and *ZmLEA5* genes. (At: *A. thaliana*; LEA: late embryogenesis-abundant; Os: *O. sativa*; Zm: *Z. mays*). Table S1: Percent similarity within and between CeLEA5s, OsLEA5s, and AtLEA5s. (At: *A. thaliana*; Ce: *C. esculentus*; LEA: late embryogenesis-abundant; Os: *O. sativa*). Table S2: *LEA_5* family genes identified in representative plant species. (AA: amino acid; Ac: *A. comosus*; Ag: *A. gramineus*; Ao: *Asparagus officinalis*; At: *A. thaliana*; Atr: *A. trichopoda*; Bd: *B. distachyon*; Bp: *B. planiculmis*; Cb: *C. breviculmis*; Ce: *C. esculentus*; Chr: chromosome; Cl: *C. littledalei*; Cr: *C. rotundus*; Cs: *C. scoparia*; Eg: *Elaeis guineensis*; Hv: *H. vulgare*; Ja: *J. ascendens*; Je: *J. effusus*; LEA: Late embryogenesis abundant; Ls: *L. sylvatica*; Pl: *P. latifolius*; Pr: *Puya raimondii*; Rb: *R. breviuscula*; Scf: scaffold; Sb: *S. bicolor*; Si: *S. italica*; Sp: *Spirodela polyrhiza*; Ss: *S. stoloniferum*; St: *S. tabernaemontani*; Tl: *T. latifolia*; WGD: whole-genome duplication; Zm: *Zea mays*). Table S3: Species-specific distribution of *LEA_5* genes in five orthogroups identified in this study. (LEA: late embryogenesis-abundant). Table S4: Primers used in this study. (Ce: *C. esculentus*; LEA: late embryogenesis-abundant).

## References

[1] Mistry J., Chuguransky S., Williams L., Qureshi M., Salazar G.A., Sonnhammer E.L.L., Tosatto S.C.E., Paladin L., Raj S., Richardson L.J. (2021). Pfam: The protein families database in 2021. Nucleic Acids Res..

[2] Cuming A.C., Lane B.G. (1979). Protein synthesis in imbibing wheat embryos. Eur. J. Biochem..

[3] Baker J., Van Dennsteele C., Dure L. (1988). Sequence and characterization of 6 Lea proteins and their genes from cotton. Plant Mol. Biol..

[4] Campos F., Cuevas-Velazquez C., Fares M.A., Reyes J.L., Covarrubias A.A. (2013). Group 1 LEA proteins, an ancestral plant protein group, are also present in other eukaryotes, and in the archeae and bacteria domains. Mol. Genet. Genomics.

[5] Artur M.A.S., Zhao T., Ligterink W., Schranz E., Hilhorst H.W. (2019). Dissecting the genomic diversification of late embryogenesis abundant (LEA) protein gene families in plants. Genome Biol. Evol..

[6] McCubbin W.D., Kay C.M., Lane B.G. (1985). Hydrodynamic and optical properties of the wheat-germ Em protein. Can. J. Biochem. Cell Biol..

[7] Russouw P.S., Farrant J., Brandt W., Lindsey G.G. (1997). The most prevalent protein in a heat-treated extract of pea (*Pisum sativum*) embryos is an LEA group I protein; its conformation is not affected by exposure to high temperature. Seed Sci. Res..

[8] Soulages J.L., Kim K., Walters C., Cushman J.C. (2002). Temperature-induced extended helix/random coil transitions in a group 1 late embryogenesis-abundant protein from soybean. Plant Physiol..

[9] Williams M.E., Tsang A. (1991). A maize gene expressed during embryogenesis is abscisic acid-inducible and highly conserved. Plant Mol. Biol..

[10] Gaubier P., Raynal M., Hull G., Huestis G.M., Grellet F., Arenas C., Pages C., Delseny M. (1993). Two different *Em*-like genes are expressed in *Arabidopsis thaliana* seeds during maturation. Mol. Gen. Genet..

[11] Stacy R.A., Espelund M., Saebøe-Larssen S., Hollung K., Helliesen E., Jakobsen K.S. (1995). Evolution of the Group 1 late embryogenesis abundant (Lea) genes: Analysis of the Lea B19 gene family in barley. Plant Mol. Biol..

[12] Wang X.S., Zhu H.B., Jin G.L., Liu H.L., Wu W.R., Zhu J. (2007). Genome-scale identification and analysis of *LEA* genes in rice (*Oryza sativa* L.). Plant. Sci..

[13] Zou Z., Huang Q.X., An F. (2013). Genome-wide identification, classification and phylogenetic analysis of *LEA* gene family in castor bean (*Ricinus communis* L.). Chin. J. Oil Crop. Sci..

[14] Zou Z., Guo J.Y., Zheng Y.J., Xiao Y.H., Guo A.P. (2022). Genomic analysis of *LEA* genes in *Carica papaya* and insight into lineage-specific family evolution in Brassicales. Life.

[15] Wu C., Hu W., Yan Y., Tie W., Ding Z., Guo J., He G. (2018). The late embryogenesis abundant protein family in cassava (*Manihot esculenta* Crantz): Genome-wide characterization and expression during abiotic stress. Molecules.

[16] Morris P.C., Kumar A., Bowles D.J., Cuming A.C. (1990). Osmotic stress and abscisic acid induce expression of the wheat *Em* genes. Eur. J. Biochem..

[17] Espelund M., Saebøe-Larssen S., Hughes D.W., Galau G.A., Larsen F., Jakobsen K.S. (1992). Late embryogenesis-abundant genes encoding proteins with different numbers of hydrophilic repeats are regulated differentially by abscisic acid and osmotic stress. Plant J..

[18] Miyoshi K., Kagaya Y., Ogawa Y., Nagato Y., Hattori T. (2002). Temporal and spatial expression pattern of the *OSVP1* and *OSEM* genes during seed development in rice. Plant Cell Physiol..

[19] Manfre A.J., Lanni L.M., Marcotte W.R. (2006). The Arabidopsis group 1 LATE EMBRYOGENESIS ABUNDANT protein ATEM6 is required for normal seed development. Plant Physiol..

[20] Manfre A.J., LaHatte G.A., Climer C.R., Marcotte W.R. (2009). Seed dehydration and the establishment of desiccation tolerance during seed maturation is altered in the *Arabidopsis thaliana* mutant *atem6-1*. Plant Cell Physiol..

[21] Vicient C.M., Hull G., Guilleminot J., Devic M., Delseny M. (2000). Differential expression of the *Arabidopsis* genes coding for Em-like proteins. J. Exp. Bot..

[22] Hundertmark M., Hincha D.K. (2008). LEA (late embryogenesis abundant) proteins and their encoding genes in *Arabidopsis thaliana*. BMC Genom..

[23] McCarty D.R., Hattori T., Carson C.B., Vasil V., Lazar M., Vasil I.K. (1991). The Viviparous-1 developmental gene of maize encodes a novel transcriptional activator. Cell.

[24] Giraudat J., Hauge B.M., Valon C., Smalle J., Parcy F., Goodman H.M. (1992). Isolation of the *Arabidopsis ABI3* gene by positional cloning. Plant Cell.

[25] Hattori T., Terada T., Hamasuna S. (1995). Regulation of the *Osem* gene by abscisic acid and the transcriptional activator VP1: Analysis of cis-acting promoter elements required for regulation by abscisic acid and VP1. Plant J..

[26] Zou Z., Xiao Y., Zhang L., Zhao Y. (2023). Analysis of *Lhc* family genes reveals development regulation and diurnal fluctuation expression patterns in *Cyperus esculentus*, a Cyperaceae plant. Planta.

[27] Zou Z., Zheng Y.J., Xiao Y.H., Liu H.Y., Huang J.Q., Zhao Y.G. (2024). Molecular insights into PIP aquaporins in tigernut (*Cyperus esculentus* L.), a Cyperaceae tuber plant. Tropical Plants.

[28] Zou Z., Fu X., Li C., Yi X., Huang J., Zhao Y. (2025). Insights into the stearoyl-acyl carrier protein desaturase (SAD) family in tigernut (*Cyperus esculentus* L.), an oil-bearing tuber plant. Plants.

[29] Makareviciene V., Gumbytea M., Yunik A. (2013). Opportunities for the use of chufa sedge in biodiesel production. Ind. Crop. Prod..

[30] Codina-Torrella I., Guamis B., Trujillo A.J. (2015). Characterization and comparison of tiger nuts (*Cyperus esculentus* L.) from different geographical origin. Ind. Crop. Prod..

[31] Yang X., Niu L., Zhang Y., Ren W., Yang C., Yang J., Xing G., Zhong X., Zhang J., Slaski J. (2022). Morpho-agronomic and biochemical characterization of accessions of tiger nut (*Cyperus esculentus*) grown in the north temperate zone of China. Plants.

[32] Yu Y., Lu X., Zhang T., Zhao C., Guan S., Pu Y., Gao F. (2022). Tiger nut (*Cyperus esculentus* L): Nutrition, processing, function and applications. Foods.

[33] Turesson H., Marttila S., Gustavsson K.E., Hofvander P., Olsson M.E., Bülow L., Stymne S., Carlsson A.S. (2010). Characterization of oil and starch accumulation in tubers of *Cyperus esculentus* var. *sativus* (Cyperaceae): A novel model system to study oil reserves in nonseed tissues. Am. J. Bot..

[34] Niemeyer P.W., Irisarri I., Scholz P., Schmitt K., Valerius O., Braus G.H., Herrfurth C., Feussner I., Sharma S., Carlsson A.S. (2022). A seed-like proteome in oil-rich tubers. Plant J..

[35] Zou Z., Zhao Y., Zhang L., Xiao Y., Guo A. (2022). Analysis of *Cyperus esculentus SMP* family genes reveals lineage-specific evolution and seed desiccation-like transcript accumulation during tuber maturation. Ind. Crop. Prod..

[36] Zou Z., Zheng Y.J., Zhang Z.T., Xiao Y.H., Xie Z.N., Chang L.L., Zhang L., Zhao Y.G. (2023). Molecular characterization *oleosin* genes in *Cyperus esculentus*, a Cyperaceae plant producing oil in underground tubers. Plant Cell Rep..

[37] Zou Z., Fu X.W., Huang J.Q., Zhao Y.G. (2024). Molecular characterization of *CeOLE6*, a diverged SH oleosin gene, preferentially expressed in *Cyperus esculentus* tubers. Planta.

[38] Zhao Y., Fu X., Zou Z. (2024). Insights into genes encoding LEA_1 domain-containing proteins in *Cyperus esculentus*, a desiccation-tolerant tuber plant. Plants.

[39] Zou Z., Zhao Y.G., Zhang L., Kong H., Guo Y.L., Guo A.P. (2021). Single-molecule real-time (SMRT)-based full-length transcriptome analysis of tigernut (*Cyperus esculentus* L.). Chin. J. Oil Crop Sci..

[40] Wang W., Haberer G., Gundlach H., Gläßer C., Nussbaumer T., Luo M.C., Lomsadze A., Borodovsky M., Kerstetter R.A., Shanklin J. (2014). The *Spirodela polyrhiza* genome reveals insights into its neotenous reduction fast growth and aquatic lifestyle. Nat. Commun..

[41] Olsen J.L., Rouzé P., Verhelst B., Lin Y.C., Bayer T., Collen J., Dattolo E., De Paoli E., Dittami S., Maumus F. (2016). The genome of the seagrass *Zostera marina* reveals angiosperm adaptation to the sea. Nature.

[42] Bredeson J.V., Lyons J.B., Oniyinde I.O., Okereke N.R., Kolade O., Nnabue I., Nwadili C.O., Hřibová E., Parker M., Nwogha J. (2022). Chromosome evolution and the genetic basis of agronomically important traits in greater yam. Nat. Commun..

[43] Emms D.M., Kelly S. (2019). OrthoFinder: Phylogenetic orthology inference for comparative genomics. Genome Biol..

[44] Jiao Y., Li J., Tang H., Paterson A.H. (2014). Integrated syntenic and phylogenomic analyses reveal an ancient genome duplication in monocots. Plant Cell.

[45] Zou Y., Wei Z., Xiao K., Wu Z., Xu X. (2023). Genomic analysis of the emergent aquatic plant *Sparganium stoloniferum* provides insights into its clonality, local adaptation and demographic history. Mol. Ecol. Resour..

[46] Schnable P.S., Ware D., Fulton R.S., Stein J.C., Wei F., Pasternak S., Liang C., Zhang J., Fulton L., Graves T.A. (2009). The B73 maize genome: Complexity, diversity, and dynamics. Science.

[47] Oliver M.J., Farrant J.M., Hilhorst H.W.M., Mundree S., Williams B., Bewley J.D. (2020). Desiccation tolerance: Avoiding cellular damage during drying and rehydration. Annu. Rev. Plant Biol..

[48] Verdier J., Lalanne D., Pelletier S., Torres-Jerez I., Righetti K., Bandyopadhyay K., Leprince O., Chatelain E., Vu B.L., Gouzy J. (2013). A regulatory network-based approach dissects late maturation processes related to the acquisition of desiccation tolerance and longevity of *Medicago truncatula* seeds. Plant Physiol..

[49] Zou Z., Zheng Y.J., Chang L.L., Zou L.P., Zhang L., Min Y., Zhao Y.G. (2024). TIP aquaporins in *Cyperus esculentus*: Genome-wide identification, expression profiles, subcellular localizations, and interaction patterns. BMC Plant Biol..

[50] Jiao Y., Leebens-Mack J., Ayyampalayam S., Bowers J.E., McKain M.R., McNeal J., Rolf M., Ruzicka D.R., Wafula E., Wickett N.J. (2012). A genome triplication associated with early diversification of the core eudicots. Genome Biol..

[51] Singh R., Ong-Abdullah M., Low E.T.L., Manaf M.A.A., Rosli R., Nookiah R., Ooi L.C.-L., Ooi S.E., Chan K.L., Azizi N. (2013). Oil palm genome sequence reveals divergence of interfertile species in Old and New worlds. Nature.

[52] Can M., Wei W., Zi H., Bai M., Liu Y., Gao D., Tu D., Bao Y., Wang L., Chen S. (2020). Genome sequence of *Kobresia littledalei*, the first chromosome-level genome in the family Cyperaceae. Sci. Data.

[53] Walters C., Ried J.L., Walker-Simmons M.K. (1997). Heat soluble proteins extracted from wheat embryos have tightly bound sugars and unusual hydration properties. Seed Sci. Res..

[54] Delahaie J., Hundertmark M., Bove J., Leprince O., Rogniaux H., Buitink J. (2013). LEA polypeptide profiling of recalcitrant and orthodox legume seeds reveals ABI3-regulated LEA protein abundance linked to desiccation tolerance. J. Exp. Bot..

[55] Hu B., Jin J., Guo A.Y., Zhang H., Luo J., Gao G. (2015). GSDS 2.0: An upgraded gene feature visualization server. Bioinformatics..

[56] Edgar R.C. (2021). MUSCLE v5 Enables Improved Estimates of Phylogenetic Tree Confidence by Ensemble Bootstrapping.

[57] Zou Z., Yang J.H. (2019). Genomic analysis of Dof transcription factors in *Hevea brasiliensis*, a rubber-producing tree. Ind. Crops Prod..

[58] Qiao X., Li Q., Yin H., Qi K., Li L., Wang R., Zhang S., Paterson A.H. (2019). Gene duplication and evolution in recurring polyploidization-diploidization cycles in plants. Genome Biol..

[59] Mortazavi A., Williams B.A., McCue K., Schaeffer L., Wold B. (2008). Mapping and quantifying mammalian transcriptomes by RNA-seq. Nat. Methods.

[60] Zou Z., Gong J., An F., Xie G.S., Wang J.K., Mo Y.Y., Yang L.F. (2015). Genome-wide identification of rubber tree (*Hevea brasiliensis* Muell. Arg.) aquaporin genes and their response to ethephon stimulation in the laticifer, a rubber-producing tissue. BMC Genom..

