# Supplementary figures and images for: Integrative Analysis Provides Insights into Genes Encoding LEA_5 Domain-Containing Proteins in Tigernut (Cyperus esculentus L.)
Source: Plants (Basel). 2025 Mar 1;14(5):762. doi: 10.3390/plants14050762 (PMC11902115; doi:10.3390/plants14050762)

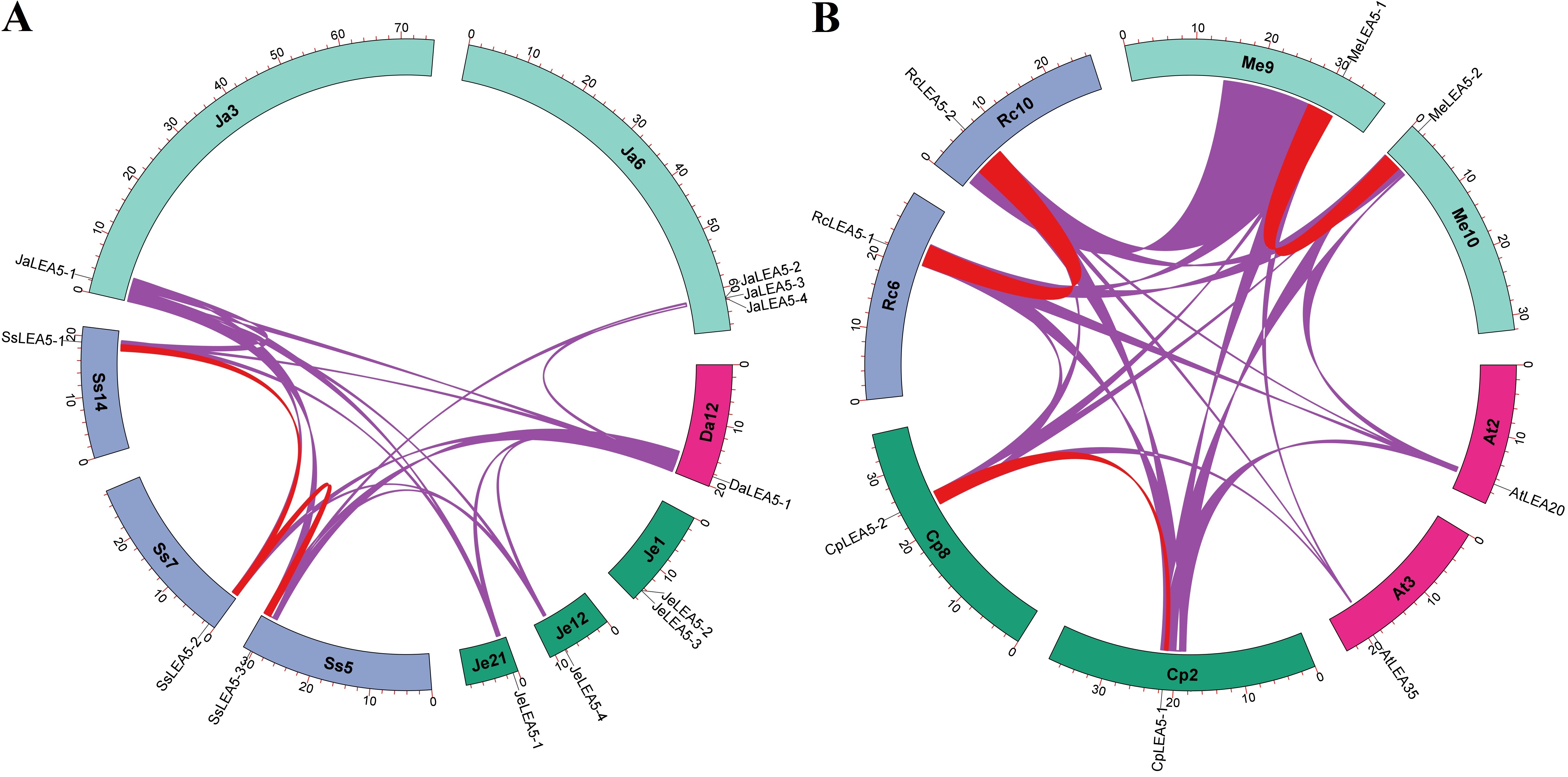

Supplement: Supplementary file 1 [file plants-14-00762-s001.zip › Figure S1.jpg]

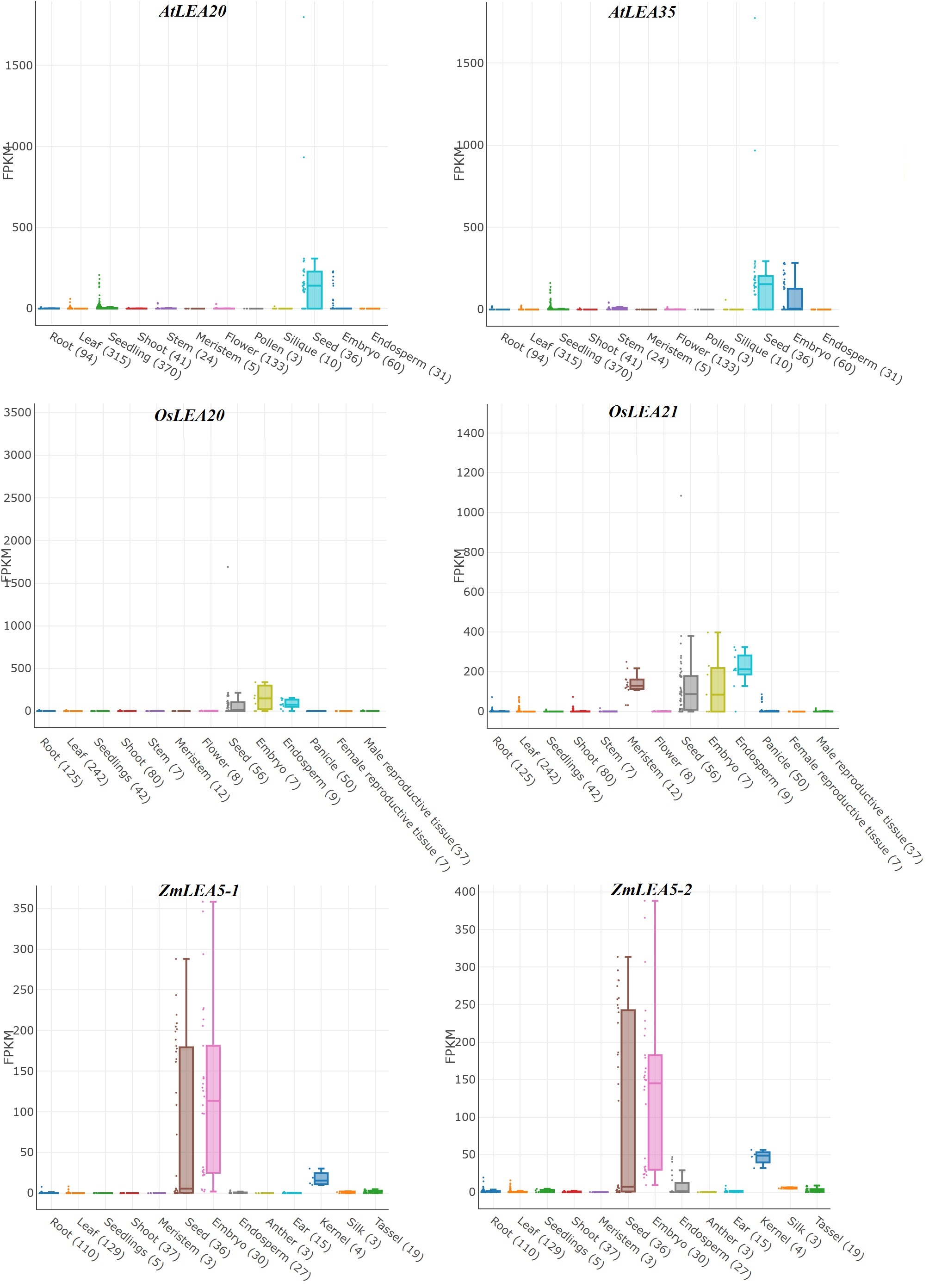

Supplement: Supplementary file 1 [file plants-14-00762-s001.zip › Figure. S2.jpg]
